# The Experimental Demonstration of High Efficiency Interaction-free Measurement for Quantum Counterfactual-like Communication

**DOI:** 10.1038/s41598-017-11305-x

**Published:** 2017-09-07

**Authors:** Chao Liu, Jinhong Liu, Junxiang Zhang, Shiyao Zhu

**Affiliations:** 1 Department of Physics, Zhejiang University, Hangzhou, 310027 P. R. China; 2 State Key Laboratory of Quantum Optics and Quantum Optics Devices, Institute of Opto-electronics, Shanxi University, Taiyuan, 030006 P. R. China; 3 Beijing Computational Science Research Center, Beijing, 100084 China; 4 Collaborative Innovation Center of Extreme Optics, Shanxi University, Taiyuan, 030006 China

## Abstract

We present an interaction-free measurement with quantum Zeno effect and a high efficiency *η* = 74.6% ± 0.15%. As a proof-of-principle demonstration, this measurement can be used to implement a quantum counterfactual-like communication protocol. Instead of a single photon state, we use a coherent light as the input source and show that the output agrees with the proposed quantum counterfactual communication protocol according to Salih *et al*. Although the counterfactuality is not achieved due to the presence of a few photons in the public channel, we show that the signal light is nearly absent in the public channel, which exhibits a proof-of-principle quantum counterfactual-like property of communication.

## Introduction

It is well known that the measurement of a quantum system inevitably destroys the quantum state unless the system is in an eigenstate of the physical observable being measured. Interaction-free-measurement (IFM)^1^ can provide a method for detecting the presence of an object without any obvious interaction. Elitzur and Vaidman proposed an IFM scheme using a Mach-Zehnder interferometer (MZI), showing that the presence of an object can be ascertained without any interaction^1^. The first experimental demonstration of the principle of IFM was performed using triggered single photon in Michelson interferometer^2^, and an enhanced efficiency was obtained due to the quantum Zeno effect^3–7^.

Based on the idea of IFM^1^, the counterfactual quantum key distribution (QKD) protocol was proposed^8^ and experimentally implemented^9, 10^, which shows that the distribution of a quantum key can be achieved even when the encoded particle does not traverse through the quantum channel. As the most practical scheme of quantum communication, QKD uses quantum principles to encode information with the quantum states of photons, ensuring that the information can be transmitted in an absolutely secure way. Any attempt of intercepting the information is likely to destroy the quantum state and be found immediately. The first quantum key distribution (QKD) protocol, known as Bennett-Brassard-84 (BB84), uses single-photon polarization states to transmit the information and was provably secure^11^. Subsequently a variety of protocols have been proposed, such as E91^12^, B92^13^. The more practical protocols of SARG04^14, 15^ were also demonstrated with weak pulses. In this scheme, the quantum key was necessarily required for preventing the photon number splitting (PNS) attack^16^ because of the probability of two or more existing photons in weak pulses. All these prominent quantum communication systems have the common feature of employing actual physical signal for information transfer. Physical transportation of quantum information may not be a viable solution for long-distance quantum communication^17^ because the interaction of the quantum system with its environment changes the quantum state. In addition the single photons may be lost while passing through the transmission channel, where the efficiency of the quantum communication will be reduced.

Recently, based on IFM and quantum Zeno effect, Salih, Li, Alamri and Zubairy (SLAZ)^18^ proposed a protocol of direct counterfactual quantum communication in a chained Mach-Zehnder set-up, in which no photon travel between Alice and Bob. This protocol is quantum mechanical as the counterfactuality is guaranteed for a single-photon input state at Alice’s end. The counterfactuality means that the probability of the existence of a photon in the public channel is strictly zero when the numbers of chained MZIs are going to be quite large. If this probability is not zero, then the protocol is not counterfactual. The experimental demonstration of the principle of counterfactual communication for SLAZ scheme with *M* = 4 and *N* = 2 was performed using a single photon source^19^. By entangling and disentangling a photon and an atom via nonlocal interaction, a new protocol for transferring an unknown quantum state counterfactually was also proposed via nonlocal interaction^20^.

An experimental implementation of the counterfactual protocol with a single photon source was usually difficult^21^, and it also requires a weak coherent light as a reference to lock the phase of the system. Therefore, the demonstration of a single-photon-based counterfactual scheme needs to be carried out with the help of a weak coherent light for phase stabilization. In this paper, we perform an experiment on high-efficiency IFM using an interlinked structure of MZIs, by this we demonstrate a proof-of-principle experiment for quantum counterfactual-like communication with coherent light. In this scheme, the quantum counterfactual property is not reached due to the presence of a few portion of light in the public channel, the scheme supplies the technology for phase stabilization of MZIs for single photon operation.

In the scheme proposed in ref. 18, a faithful interaction-free measurement system can be obtained with multiple MZIs e.g. there are *M* − 1 outer MZIs (i.e., there are *M* beamsplitters in these MZIs) connected in series, while there are *N* − 1 small MZIs (i.e. *N* beamsplitters are included) connected in series in one arm of each outer MZI. An efficiency approaching 100% can be realized if *M* and *N* are large enough. However, for a practical setup, the inevitable loss resulting from the optical elements will be introduced and it will increase with the number of the MZIs. As a result, the efficiency will decrease correspondingly. One must have a balanced consideration for accomplishing this protocol with a proper number of MZI.

In our experimental setup, the structure of MZIs is designed with a multiple-series connection, consisting of two outer MZIs and seven inner MZIs in one arm of each outer MZI. The reflectivity of the beamsplitters in MZIs is specially designed to satisfy the effect of IFMs, which gives the possibility of detecting the presence of an object without direct interaction with the object. The detection of the intensity ratio of the two final outputs can also tells the possible operation of quantum counterfactual-like communication in principle.

## Results

### Interaction-free measurement with two outputs for quantum counterfactual-like communication

**Figure 1:**
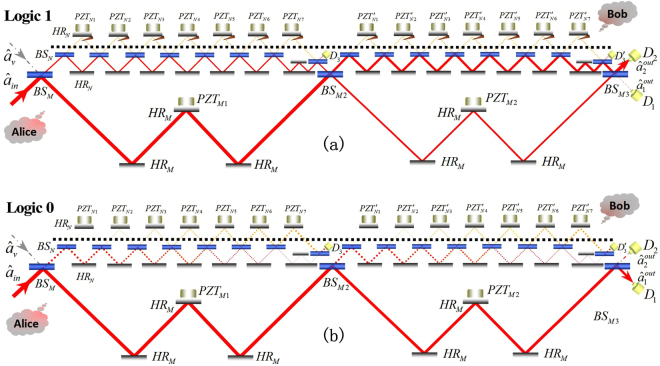
The schematic diagram of the experiment. Weak coherent light represented by the annihilation operator ${\hat{a}}_{in}$ and vacuum state ${\hat{a}}_{v}$ is injected into the other input port. There are four outputs (${\hat{a}}_{3}^{out}$, ${\hat{a}}_{3^{\prime} }^{out}$, ${\hat{a}}_{1}^{out}$ and ${\hat{a}}_{2}^{out}$) detected by the photoelectric detectors (D_3_, ${{\rm{D}}}_{3}^{^{\prime} }$, D_1_ and D_2_). Blocks are indicated by triangles in the transmission channel. HR_*M*,*N*_: High reflection mirrors; BS_*M*1−*M*3,*N*_: Beamsplitter; PZT_*M*1,*M*2_ (${{\rm{PZT}}}_{N1-N7}^{^{\prime} }$): Piezoelectric transducer. The experimental setup (**a**) Logic 1 with block and (**b**) Logic 0 without block.

We design the experiment with *M* = 3 (i.e two big MZIs) and *N* = 8 (i.e. seven small MZIs), as illustrated in Fig. 1. The number of MZIs is optimized to get the maximum outputs when considering the possible loss, the theoretical discussion is given below. Two outer MZIs are connected in series. In each of the two big MZIs, there are seven small MZIs connected in series. Thereforehree beam splitters BS_*Mj*(*j*=1,2,3)_ in both the MZIs are designed with the same reflectivity ${R}_{M}={\cos }^{2}\,(\pi \mathrm{/6)}$, corresponding to the experimental coating of 75% ± 1%. In addition, sixteen (2 × 8) beam splitters BS_*N*_ in small MZIs have the reflectivity ${R}_{N}={\cos }^{2}\,(\pi \mathrm{/16)}$, with the experimental value being 96.2% ± 0.5%. The mirrors HR_*M*_ and HR_*N*_ are high reflection mirrors with reflectivity larger than 99.99%. The wavelength band selection for the optics in our setup is 790 nm~950 nm since the wavelength of light source is 850 nm, and each of the optics are coated for horizontally polarized light. To extend the scheme in C + L wavelength band, one can simply change the light source and optics for suitable wavelength.

The piezoelectric transducers (PZT_*M*1,*M*2_, PZT_*Ni*(*i*=1,…,7)_ and ${{\rm{PZT}}}_{Ni(i=1,\ldots \mathrm{,7)}}^{^{\prime} }$) are used to adjust the path difference (the phase difference is set to be 2*nπ*) of each MZI. D_3_, ${{\rm{D}}}_{3}^{^{\prime} }$, D_1_ and D_2_ are the photon detectors with the same sensitivity and 85% quantum efficiency at 852 nm wavelength. A 490 *μ*W coherent light represented by the annihilation operator ${\hat{a}}_{in}$ is incident into the system through the first beamsplitter (BS_*M*1_), while the other input port of BS_*M*1_ is in vacuum represented by the operator ${\hat{a}}_{v}$. There are four outputs represented by the operators ${\hat{a}}_{3}^{out}$, ${\hat{a}}_{3^{\prime} }^{out}$, ${\hat{a}}_{1}^{out}$ and ${\hat{a}}_{2}^{out}$ corresponding to the detectors by D_3_, ${{\rm{D}}}_{3}^{^{\prime} }$, D_1_ and D_2_, respectively. Fourteen (2 × 7) blocks indicated by triangles in one arm (as the transmission channel) of each small MZIs are served as switch (or logic gate) to let the light be absorbed by the blocks (corresponding to logic 1 in Fig. 1a) or pass through the MZIs (logic 0 in Fig. 1b).

In order to create the communication between Alice and Bob, this system is assigned to have two separated parts at Alice’s and Bob’s sides. The input light (represented by annihilation operator ${\hat{a}}_{in}$), the detectors and optics below the black dash line are at Alice’s side, while the blocks and optics above the black dashed line are at Bob’s side. Bob’s selection of Logic 1 or 0 leads the input light to detectors D_2_ or D_1_, i.e. communication is created between Alice and Bob. In order to have direct counterfactual quantum communication, almost no light should pass through the public or transmission channel (see the yellow line in Fig. 1).

We use the transfer-matrix method to verify the propagation of the input light of the system. If we denote the input light by the column vector ${{\boldsymbol{(}}{\hat{a}}_{in},{\hat{a}}_{v}{\boldsymbol{)}}}^{{\rm{T}}}$, the output light can be written as

1 $$(\begin{array}{c}{\hat{a}}_{1}^{out}\\ {\hat{a}}_{2}^{out}\end{array})={T}_{t(logic)}\,(\begin{array}{c}{\hat{a}}_{in}\\ {\hat{a}}_{v}\end{array})$$

The transformation *T*_*t*(*logic*)_ takes the different forms for two cases of logic 1 and logic 0. We consider the two cases separately.

- **Logic 1** For the case of logic 1 in Fig. 1a with absorbers at the Bob’s ends in all (*N* − 1) × (*M* − 1) MZIs, the transfer matrix of BS_*Mj*(*j*=1,2,3)_ is described as
  2 $${T}_{B{S}_{M}}=(\begin{array}{cc}{r}_{m} & -{t}_{m}\\ {t}_{m} & {r}_{m}\end{array}),$$
  where *r*_*m*_ = cos *π*/2*M*, *t*_*m*_ = sin *π*/2*M* are the reflection and transmission amplitudes of the BS_*Mj*_. The reflection matrix of “*N*” BS_*N*_ is described as
  3 $${W}_{Ni}=(\begin{array}{cc}1 & 0\\ 0 & \cos \,\frac{\pi }{2N}\end{array}),$$
  and the effect of the phase difference *φ*_*j*_ via the HR_*M*_ is represented by
  4 $${W}_{Mj}=(\begin{array}{cc}{e}^{i{\phi }_{j}} & 0\\ 0 & 1\end{array})\,(j=1,\ldots ,M-1).$$
  Finally, the total transfer matrix is expressed as
  5 $${T}_{t1}={({T}_{B{S}_{M}}\cdot {W}_{Mj}\cdot {({W}_{Ni})}^{N})}^{M-1}\cdot {T}_{B{S}_{M}}=(\begin{array}{cc}{A}_{11} & {A}_{12}\\ {A}_{21} & {A}_{22}\end{array}),$$
  here *A*_*lk*_(*l*, *k* = 1, 2) are the matrix elements of *T*_*t*1_.
  The output intensity *I*(D_1_) and *I*(D_2_) at detectors D_1_ and D_2_ are given by ${\hat{a}}_{1}^{out\dagger }{\hat{a}}_{1}^{out}={A}_{11}^{\ast }{A}_{11}{\hat{a}}_{in}^{\dagger }{\hat{a}}_{in}$, ${\hat{a}}_{2}^{out\dagger }{\hat{a}}_{2}^{out}={A}_{21}^{\ast }{A}_{21}{\hat{a}}_{in}^{\dagger }{\hat{a}}_{in}$. In Table 1, we present the theoretical calculation of intensity ratio *I*(D_2_)/*I*(D_1_) for different choices of *N* and *M* when the phase differences of big MZIs are *φ*_*j*_ = 0 or 2*nπ*. We note that most of the light exits via the output port at D_2_ due to constructive interference, while a small intensity of light is detected at port D_1_ due to destructive interference. We also note that the higher ratio is obtained when the number *M* of big MZIs is smaller than the number *N* of small MZIs, i.e., *M* < *N*. According to this discussion, we take *M* = 3, *N* = 8 in the experiment as shown in Fig. 1.

**Table 1:** The theoretical intensity ratio of *I*(D_2_)/*I*(D_1_) for Logic 1.

	*N* = 2	*N* = 3	*N* = 4	*N* = 5	*N* = 6	*N* = 7	*N* = 8
*M* = 2	9	22.15	40.55	64.20	93.11	127.28	166.70
*M* = 3	2.46	6.31	11.91	19.26	28.36	39.21	51.81
*M* = 4	1.09	2.84	5.47	8.99	13.41	18.73	24.94
*M* = 5	0.6 < 1	1.56	3.04	5.06	7.62	10.73	14.40
*M* = 6	$\ll$1	0.96 < 1	1.89	3.17	4.81	6.83	9.21
*M* = 7	$\ll$1	$\ll$1	0.96 < 1	2.13	3.26	4.65	6.31
  In Figs 2 and 3, we plot the theoretical and experimental interference fringes of each of the two big MZIs for *N* = 8, *M* = 3, in which, the solid lines (constructive) and dashed line (destructive) are obtained at outputs of D_2_ and D_1_, respectively. The blue and black lines are for the results of first (with the second big MZI locked) and second big MZI (with the first big MZI locked), respectively.

**Figure 2:**
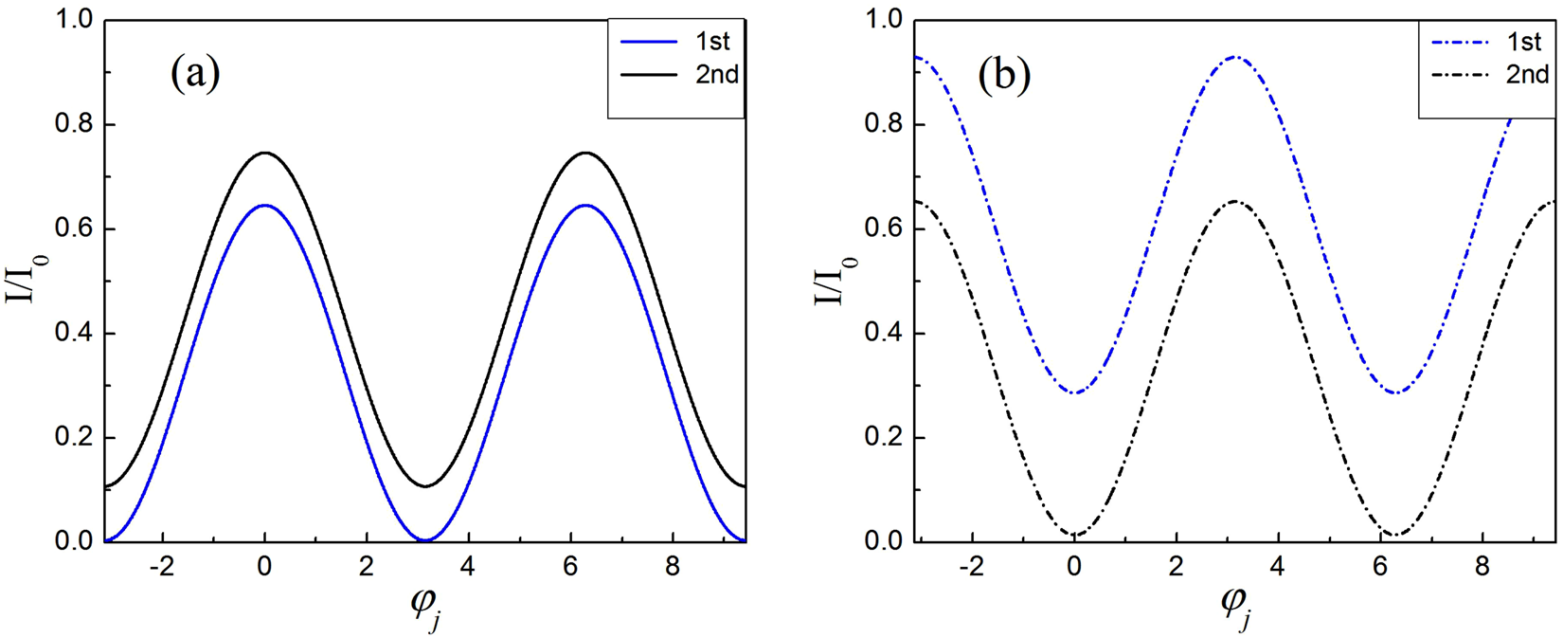
The theoretical interference fringe of each two big MZIs for Logic 1. The theoretical (**a**) constructive (solid lines) interference fringes from bottom to top and (**b**) destructive (dashed lines) interference fringes from top to bottom. The blue (the second big MZI locked) and black (the first big MZI locked) lines are for the results of first and second big MZI respectively.

**Figure 3:**
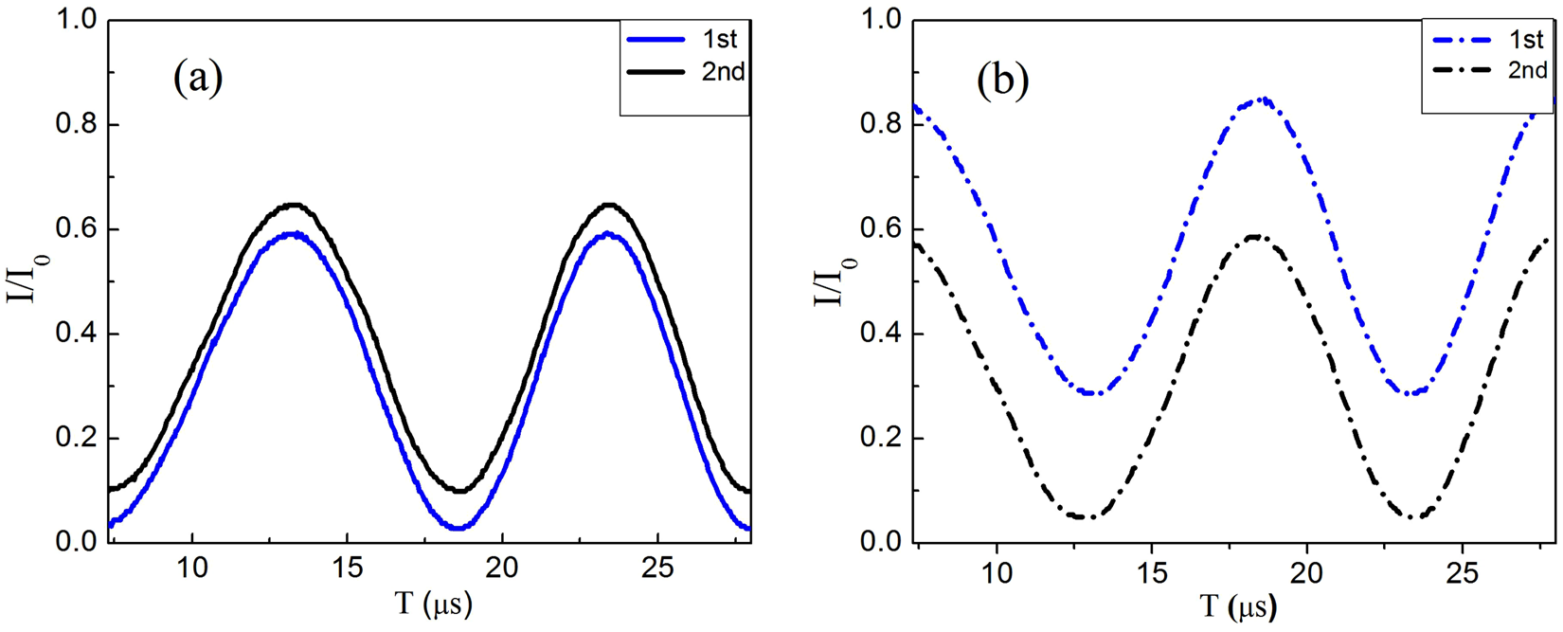
The experimental interference fringe of each two big MZIs for Logic 1. The experimental (**a**) constructive (solid lines) interference fringes and (**b**) destructive (dashed lines) interference fringes.
  In the experiment, the intensity ratio of *I*(D_2_)/*I*(D_1_) is obtained when we lock the phase differences of two big MZIs to be *φ*_1_ = *φ*_2_ = 0. During the locking time, the stable intensities at D_2_ (solid line) and D_1_ (dashed line) as a function of the time are obtained as shown in Fig. 4, corresponding to the normalized intensities *I*(D_2_)/*I*_0_ = 65% at D_2_ and *I*(D_1_)/*I*_0_ = 5% at D_1_ respectively, where *I*_0_ is the initial light intensity. It gives the ratio of *I*(D_2_)/*I*(D_1_) = 13.0, which is lower than the theoretical predication of 51.81 (see Table 1) with *I*(D_2_) = 74.6% and *I*(D_1_) = 1.44%. The deviation mainly comes from the loss in the arm with 2*N* BS_*N*_, leading to the unbalanced intensity of light in two arms of big MZIs, as a result decrease in the visibility of fringes. Note that, in order to lock the phase of the two big MZIs, we inject a relative strong reference light from the vacuum input part, the first MZI is locked using the interference fringe at the upper output from the first HR_*M*_ at the second big MZI, while the second MZI is locked using the interference fringe at D_1_. The measurement is carried on untill the reference light beam and the Lock-in-system switch off via computer controlled devices.

**Figure 4:**
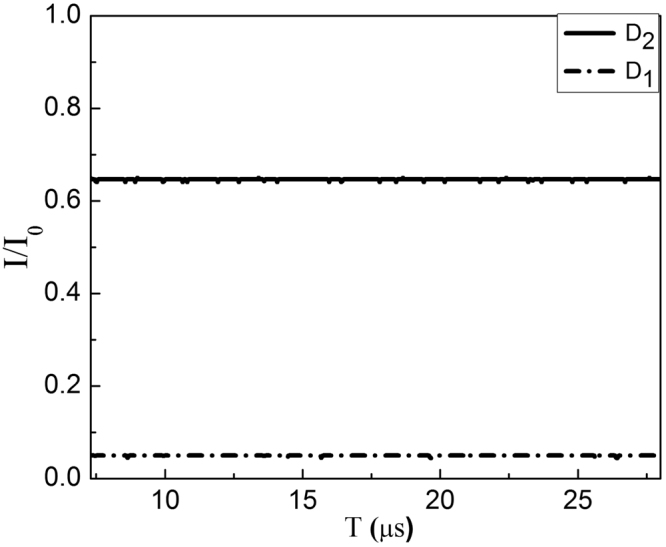
The detected normalized intensity when the phase is locked for Logic 1. The stable intensities of D_2_ (solid line) and D_1_ (dashed line) when locking the phase differences of two big MZIs to be *φ*_1_ = *φ*_2_ = 0, giving the result *I*(D_2_)/*I*_0_ = 65% and *I*(D_1_)/*I*_0_ = 5%.
- **Logic 0** Next we consider the case of logic 0 in Fig. 1b without blocks at Bob’s end. First let us discuss the array of *N* − 1 small MZIs in the inner loops. Once the input light enter the small MZIs, the constructive interference makes the light exit at the ports of detectors D_3_, ${{\rm{D}}}_{3}^{^{\prime} }$ (see the black line in Fig. 5a at *ϕ*_*i*_ = 0), while the destructive interference as a result no light enters the big MZIs from the small MZIs (see the black line in Fig. 5b at *ϕ*_*i*_ = 0).

**Figure 5:**
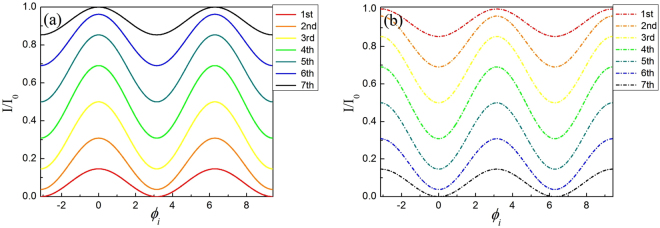
The theoretical interference fringes of the outputs of each small group of MZIs for logic 0. The seven curves (**a**) from bottom to top and (**b**) from top to bottom are the theoretical constructive and destructive interferences for the outputs of the seven MZIs in one small MZIs group.

The transfer matrix of BS_*N*_ is described as

6 $${T}_{B{S}_{N}}=(\begin{array}{cc}{r}_{n} & -{t}_{n}\\ {t}_{n} & {r}_{n}\end{array}),$$

where *r*_*n*_ = cos *π*/2*N*, *t*_*n*_ = sin *π*/2*N* are the reflection and transmission amplitudes of the BS_*N*_, respectively. the effect of the phase difference *ϕ*_*i*_ via the HR_*N*_ can be represented as

7 $${W}_{Ni}^{^{\prime} }=(\begin{array}{cc}{e}^{i{\varphi }_{i}} & 0\\ 0 & 1\end{array})\,(i=1,\ldots ,N-1),$$

therefore, we can consider the transfer matrix for small MZIs as

8 $${T}_{N}={({T}_{B{S}_{N}}\cdot {W}_{Ni}^{^{\prime} })}^{N-1}\cdot {T}_{B{S}_{N}}=(\begin{array}{cc}{C}_{11} & {C}_{12}\\ {C}_{21} & {C}_{22}\end{array}),$$

here *C*_*lk*_(*l*, *k* = 1, 2) are the matrix elements of *T*_*N*_.

**Figure 6:**
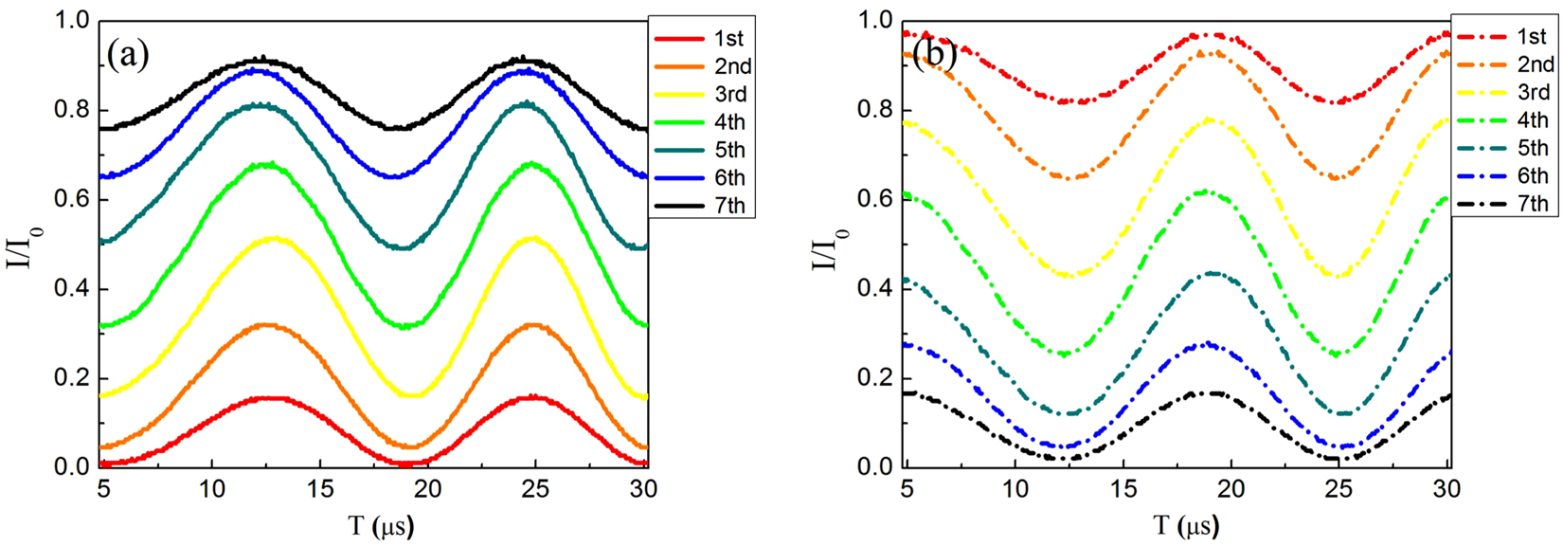
The experimental interference fringes of the outputs of each small group of MZIs for logic 0. The experimental (**a**) constructive (solid lines) and (**b**) destructive (dashed lines) interference at the output ports of each small MZI in the first MZIs group, and detected by D_3_ is 91.7%.

The theoretical and experimental interferences are plotted in Figs 5 and 6, respectively. The solid lines (constructive) would refer the to detection at D_3_ and the dashed lines (destructive) would refer to the detection before entering a big MZI. The seven curves from bottom to top in Figs 5a and 6a are the theoretical and experimental constructive interferences for the seven MZIs in the first small MZIs group, respectively. The corresponding curves in Figs 5b and 6b are the theoretical and experimental curves for the destructive interferences for the outputs of the seven MZIs in the first small MZIs group. Note that the measurement of each interference is done with the condition that the (*N* − 1)th fringe is obtained when the phase of the other small MZIs is *ϕ*_*i*_ = 0.

When all the seven small MZIs work with the phase difference *ϕ*_*Ni*_ = 0(*i* = 1, …, 7), the coherent state will completely exit to D_3_. Theoretically, we should have *I*(D_3_) = *I*(${{\rm{D}}}_{3}^{^{\prime} }$) = 100% without loss and dissipation. Here due to the dissipation, non-perfect mirrors and loss of other optical elements, the experimental value for the normalized intensity detected at D_3_ is 91.7%, as shown in Fig. 6a. Almost the same result is obtained for the detection at ${{\rm{D}}}_{3}^{^{\prime} }$, which is 91.5%. Experimentally, we find the intensity leakage from the small MZIs back to the second big MZI (from last *BS*_*N*_ to *BS*_*M*3_) approximately 0.7% of the intensity just before the first *BS*_*N*_.

Note that the measurement of each interference is done with the condition that the (*N* − 1)th fringe is obtained when the phase of the other small MZIs is *ϕ*_*i*_ = 0, as shown in Fig. 6. The phase is controlled via a computer controlled series of lock-in systems. This process is similar with the lock-in system for two big MZIs, we inject a strong reference beam from vacuum input part, then with the aid of the interference fringes from upper HR_*N*_s, HR_*M*_s and D_1_, the interlinked MZIs are locked. Thereafter, we switch off the injected beam and lock-in system to detect the results.

After finishing the adjustment of the small chains of MZIs, we consider the final output intensity *I*(D_1_) and *I*(D_2_) at detectors D_1_ and D_2_ for the case of logic 0, that is ${\hat{a}}_{1}^{out\dagger }{\hat{a}}_{1}^{out}={G}_{11}^{\ast }{G}_{11}{\hat{a}}_{in}^{\dagger }{\hat{a}}_{in}$, ${\hat{a}}_{2}^{out\dagger }{\hat{a}}_{2}^{out}={G}_{21}^{\ast }{G}_{21}{\hat{a}}_{in}^{\dagger }{\hat{a}}_{in}$. Here *G*_*lk*_(*l*, *k* = 1, 2) are the matrix elements of *T*_*t*0_, given by

9 $${T}_{t0}={({T}_{B{S}_{M}}\cdot {W}_{Mj}^{^{\prime} })}^{M-1}\cdot {T}_{B{S}_{M}}=(\begin{array}{cc}{G}_{11} & {G}_{12}\\ {G}_{21} & {G}_{22}\end{array}).$$

The theoretical value of *I*(D_1_)/*I*(D_2_) = 3 is obtained when we set the phase difference *φ*_*j*_ = 2*nπ* in matrix:

10 $${W}_{Mj}^{^{\prime} }=(\begin{array}{cc}{e}^{i{\phi }_{j}} & 0\\ 0 & 0\end{array}).$$

**Figure 7:**
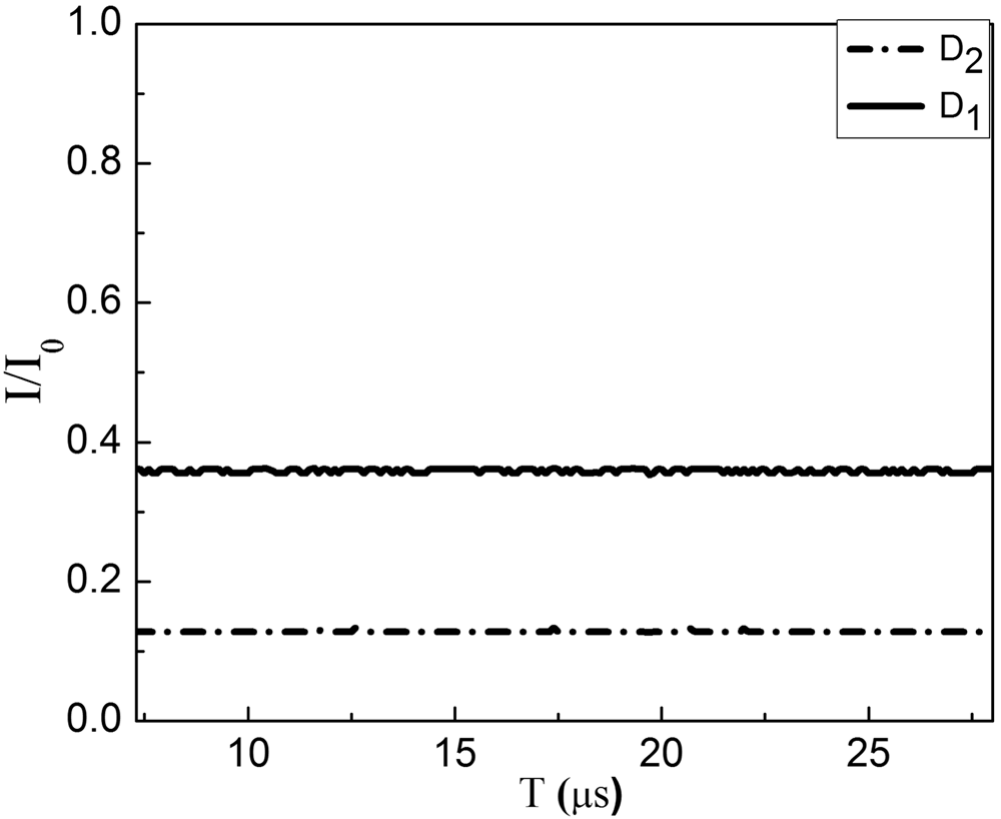
The detected normalized intensity for logic 0. The stable intensities at D_1_ (solid line) and D_2_ (dashed line) when locking the phase differences of two big MZIs to be *φ*_1_ = *φ*_2_ = 0, giving the result *I*(D_1_)/*I*_0_ = 36% and *I*(D_2_)/*I*_0_ = 13%.

Experimentally when we set *φ*_*j*_ = 0 via the PZT on the HR_*M*_, the stable intensities at D_1_ (solid line) and D_2_ (dashed line) are obtained as a function of time as shown in Fig. 7. The normalized intensity is 36% at D_1_ and 13% at D_2_, corresponding to the ratio *I*(D_1_)/*I*(D_2_) = 2.77. Once the two groups of small MZIs works, the constructive interference makes most of the light coming out of the outer MZIs, or a few partial light is maintained in the path of the big MZIs, therefore the detection values of *I*(D_1_)/*I*(D_2_) is mainly determined by the reflection of BS_*M*1_ and both reflection and transmission of BS_*M*2_. This results in the small violation between the theoretical and experimental values as a consequence of the inevitable losses of BS_*M*1,2_ and light transmission in the path of MZIs.

### The efficiency of interaction-free measurement

For the two cases, with block and without block, the experimental results which we had measured are *I*(D_2_)/*I*(D_1_) = 13.0 for logic 1 and *I*(D_1_)/*I*(D_2_) = 2.77 for logic 0. For logic 1, most of light comes throughout the output port at D_2_, on the contrary for logic 0, most of light comes throughout the output port at D_1_. Therefore, Alice can read the information of Bob’s logic gates by measuring the intensities at the two detectors, D_1_ and D_2_.

It is important to note that, although the information of logic encoded in the light of transmission channel (yellow line) is inferred from the detections at D_1_ and D_2_, the light in the transmission channel actually do not reach to the outputs of D_1_ and D_2_, it goes out from the output port at D_3_ and ${{\rm{D}}}_{3}^{^{\prime} }$. The results demonstrate, in principle, that the quantum communication could be realized via interaction-free measurement of quantum logic. It also shows that few light is transferring through the transmission channel, proving the idea of quantum counterfactual-like communication to be accessible.

**Figure 8:**
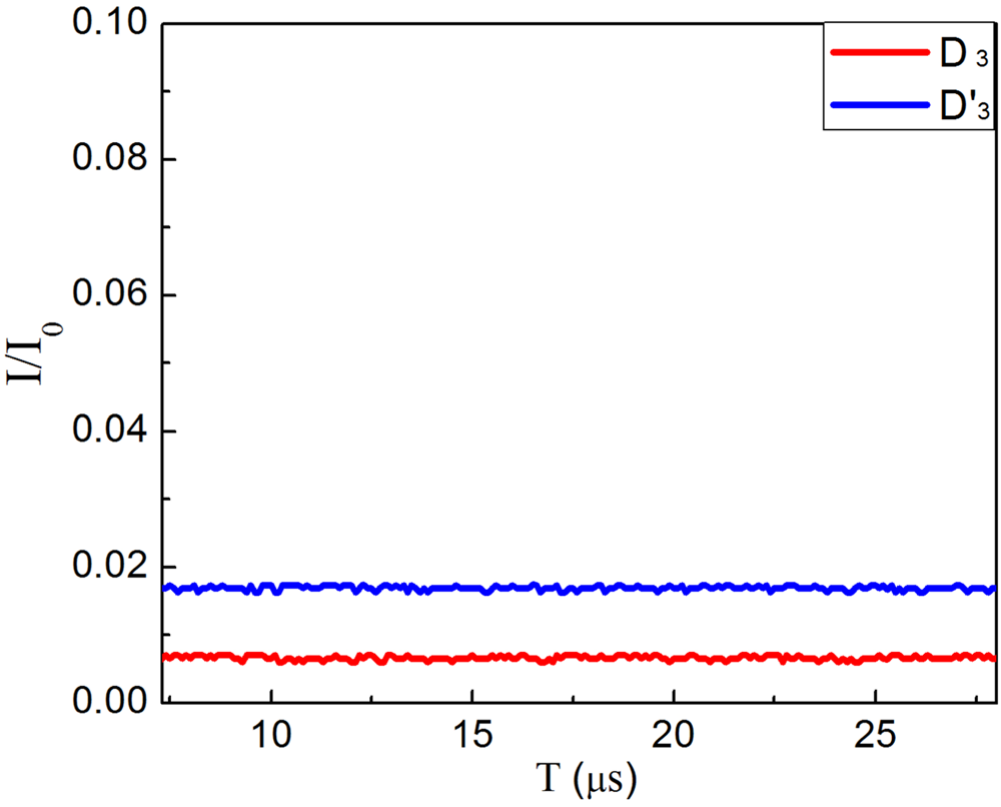
The detected normalized intensity of the detectors D_3_ and ${{\rm{D}}}_{3}^{^{\prime} }$ for logic 1. The stable intensities at D_3_ (red line) and ${{\rm{D}}}_{3}^{^{\prime} }$ (blue line) for logic 1. giving the result *I*(D_3_)/*I*_0_ = 0.7% and *I*(${{\rm{D}}}_{3}^{^{\prime} }$)/*I*_0_ = 1.7%.

Our experiment is the realization of interaction-free measurement with a large efficiency. We note that when light enters the small MZIs for logic 1, it exits from the output port of D_3_ or ${{\rm{D}}}_{3}^{^{\prime} }$ which is 0.7% and 1.7% respectively as shown in Fig. 8 (note D_3_ and ${{\rm{D}}}_{3}^{^{\prime} }$ are in the hands of Alice), which is not detected by the detectors D_1_ and D_2_ (in the hands of Alice). This process corresponds to interaction-free measurement, which is clearly seen from Eq. (5) and evident from Figs 2 and 3. For our experimental system, we use the fraction *η*, defined by *η* = *P*_*det*_/(*P*_*det*_ + *P*_*abs*_) in ref. 2, to characterize the quality of the interaction-free measurement. Here *P*_*det*_ is the probability of an interaction-free measurement and *P*_*abs*_ is the probability that the light is absorbed by the block (including other loss factors). For a perfect interaction-free measurement, we have *P*_*abs*_ = 0, i.e. *η* = 1. The range 0 < *η* < 1 represents the system which accomplishes interaction-free measurement with finite efficiency, e.g., *η* = 1/2 in ref. 1, *η* = 2/3 in ref. 2.

For our scheme, *η* is obtained from the evolution of the input state $|{a}_{in}\rangle$

11 $$|{a}_{in}\rangle \to \alpha |{a}_{1}^{out}\rangle +\beta |{a}_{2}^{out}\rangle +\sum _{l=0}^{N-1}\,({\mu }^{l}+{\nu }^{l})\,|abs\rangle ,$$

where $\alpha ={r}_{m}({r}_{m}^{2}-{t}_{m}^{2}{r}_{n}^{N})-{r}_{m}{t}_{m}^{2}{r}_{n}^{N}\mathrm{(1}+{r}_{n}^{N})$, $\beta =-{t}_{m}({r}_{m}^{2}-{t}_{m}^{2}{r}_{n}^{N})-{r}_{m}^{2}{t}_{m}{r}_{n}^{N}\mathrm{(1}+{r}_{n}^{N})$, *μ* ^*l*^ = *t*_*n*_ *t*_*m*_(*r*_*n*_)^*l*^, ${\nu }^{l}={r}_{m}{t}_{m}{t}_{n}\mathrm{(1}+{r}_{n}^{N})\,{({r}_{n})}^{l}$. Here $|{a}_{1}^{out}\rangle$ and $|{a}_{2}^{out}\rangle$ are the output states at the detectors D_1_ and D_2_, and $|abs\rangle$ represents the absoption by the block in the transmission channel. Therefore we have

12 $${P}_{det}={\beta }^{2},\,{P}_{abs}=\sum _{l=0}^{N-1}\,({\mu }^{2l}+{\nu }^{2l}).$$

**Figure 9:**
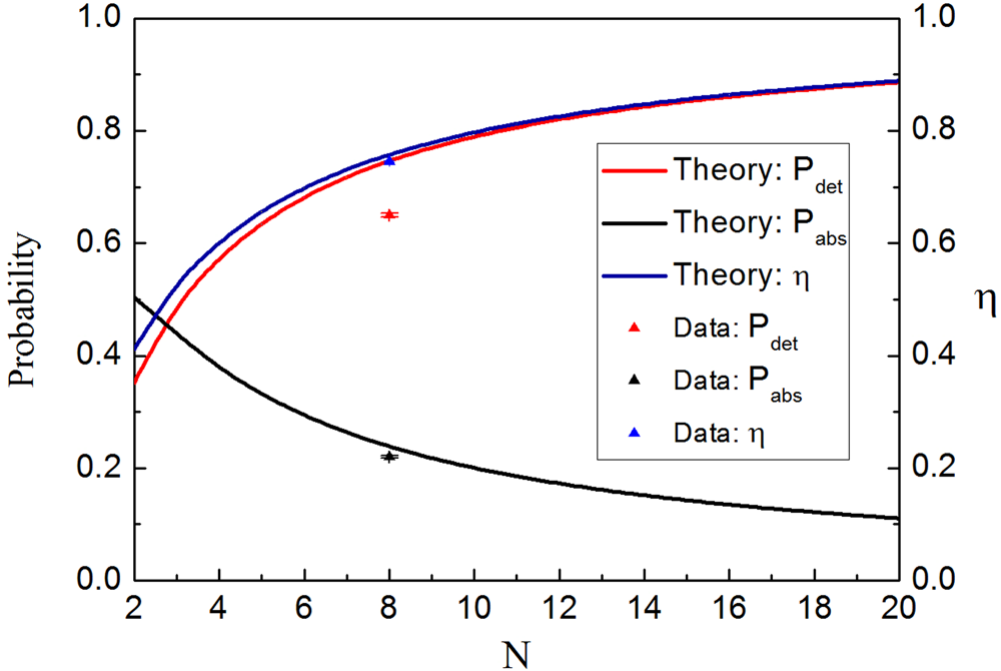
The probability of *P*_*det*_, *P*_*abs*_ and *η* vs number *N* for *M* = 3. The solid lines are the calculated probability of *P*_*det*_, *P*_*abs*_ and *η* when *N* is selected as 2, …, 20 with the number of *M* = 3. The triangle points are the experimentally measured results when *N* = 8.

In Fig. 9, The solid lines show the dependence of probability *P*_*det*_ and *P*_*abs*_ on *N* with the number of *M* = 3 for the case of logic 1. It is shown that *P*_*det*_ increases and *P*_*abs*_ decreases with the increase of *N*. As a result, the efficiency *η* increases when *N* increases, it approaches complete interaction free when *N* → ∞. The triangle points show the experimental results for *P*_*det*_, *P*_*abs*_ and *η* with *M* = 3, *N* = 8 for logic 1. *P*_*det*_ represents the detection of light probability at D_2_, and *P*_*det*_ = *P*(D_2_) = 65% is obtained from the experimental data in Fig. 3a for the maximum value, while *P*_*abs*_ is the probability of light absorbed or lossed by all the mirrors above the black dashed line, it is read from the detectors D_3_, ${{\rm{D}}}_{3}^{^{\prime} }$ in Fig. 8 according to the calculation of ${P}_{abs}={\sum }_{l=0}^{7}\,[(P({{\rm{D}}}_{3})+P({{\rm{D}}}_{3}^{^{\prime} }))/{r}_{n}^{2l}]=\mathrm{22 \% }$. Note that, in this case, *P*(D_1_) = 5% (see Fig. 3b), the total loss of the system induced by the mirrors and other elements is the 8%. Finally, we obtain the values for our system with *N* = 8, *M* = 3, corresponding to *η* = 0.76 for theory and *η* = 74.6% ± 0.15% for experiment in repeated measurements, which is larger than the predicted and reported results of *η* = 1/2 in refs 1 and 2 or improved value of *η* = 2/3 in ref. 2.

## Discussion

In conclusion, we performed an experiment of the high-efficiency interaction-free measurement. Based on the measurement, the quantum counterfactual-like communication with few portion of light involved in the transmission channel can be reached in this scheme. we analysed and implemented a principle scheme with finite *M* and *N* of linked interferometers, in which the inevitable loss of optics is involved and increased with the increases of the number of *M* and *N*, causing an unexpected decrease of the quantum efficiency of the system. We showed that the practical scheme of high-efficiency interaction-free measurement with low number of *M* and relatively higher number *N* is accessible.
